# Improved EEG-based emotion recognition through information enhancement in connectivity feature map

**DOI:** 10.1038/s41598-023-40786-2

**Published:** 2023-08-23

**Authors:** M. A. H. Akhand, Mahfuza Akter Maria, Md Abdus Samad Kamal, Kazuyuki Murase

**Affiliations:** 1 Department of Computer Science and Engineering, Khulna University of Engineering & Technology, Khulna, 9203 Bangladesh; 2 Graduate School of Science and Technology, Gunma University, Kiryu, 376-8515 Japan; 3 Department of Information Technology, International Professional University of Technology in Osaka, 3-3-1 Umeda, Kita-ku, Osaka, 530-0001 Japan

**Keywords:** Biomedical engineering, Emotion

## Abstract

Electroencephalography (EEG), despite its inherited complexity, is a preferable brain signal for automatic human emotion recognition (ER), which is a challenging machine learning task with emerging applications. In any automatic ER, machine learning (ML) models classify emotions using the extracted features from the EEG signals, and therefore, such feature extraction is a crucial part of ER process. Recently, EEG channel connectivity features have been widely used in ER, where Pearson correlation coefficient (PCC), mutual information (MI), phase-locking value (PLV), and transfer entropy (TE) are well-known methods for connectivity feature map (CFM) construction. CFMs are typically formed in a two-dimensional configuration using the signals from two EEG channels, and such two-dimensional CFMs are usually symmetric and hold redundant information. This study proposes the construction of a more informative CFM that can lead to better ER. Specifically, the proposed innovative technique intelligently combines CFMs’ measures of two different individual methods, and its outcomes are more informative as a fused CFM. Such CFM fusion does not incur additional computational costs in training the ML model. In this study, fused CFMs are constructed by combining every pair of methods from PCC, PLV, MI, and TE; and the resulting fused CFMs PCC + PLV, PCC + MI, PCC + TE, PLV + MI, PLV + TE, and MI + TE are used to classify emotion by convolutional neural network. Rigorous experiments on the DEAP benchmark EEG dataset show that the proposed CFMs deliver better ER performances than CFM with a single connectivity method (e.g., PCC). At a glance, PLV + MI-based ER is shown to be the most promising one as it outperforms the other methods.

## Introduction

The process of identifying the mental states or conditions of the human mind is known as emotion recognition (ER), and the brain signal is the most prospectus for ER. ER has become an essential part of research in neurology, computer science, cognitive science, and medical science^1^. Most commonly, modalities such as facial images^2^, speech^3^, and gestures^4^ can be used to identify emotions. However, these approaches to recognition are not ubiquitous and have low recognition accuracy because they depend on the person’s age, appearance, culture, language, and habits^5^. On the other hand, the brain is regarded as the place where emotional activities are evoked^6^. According to research in cognitive psychology and neuropsychology, the development and evolution of emotions are strongly related to the functioning of the central nervous system^7^. Therefore, the brain signal is highly reliable for ER. Among different brain signals, Electroencephalography (EEG) signal has drawn great attention in the emotion recognition research community.

EEG can capture the electrical impulses generated by neuronal activities of the brain through its small sensors (i.e., EEG channels) attached to the brain; it records the voltage alterations caused by ionic current flows within the brain’s neurons^8^. Recently, EEG has been studied for different applications in real-life human activities, such as emotion recognition^9^, autism detection^10^, recognition of seizures^11^, depression detection^12^, motor imagery classification^13^, and so on. EEG has been increasingly popular for researching the brain’s reactions to emotional stimuli and responses for its excellent temporal resolution, noninvasiveness, portability, ease of use, and relatively inexpensive and fast^7,8^. EEG is a composite signal, and different mental states are incorporated into the various EEG sub-bands of waves. Delta (0–3 Hz), Theta (4–7 Hz), Alpha (8–12 Hz), Beta (13–29 Hz), and Gamma (30–50 Hz) are the five main types of brain waves that make up the human EEG. These sub-bands may provide more precise information on the constituent neuronal processes activities^14,15^. Different mental states and activities are incorporated into the various sub-bands. Emotion has a strong relationship with the Gamma and Beta sub-bands, a weak relationship with the Alpha sub-band, and a very weak relationship with the Theta and Delta sub-bands^16^.

The typical steps of EEG-based ER are extracting features from EEG signals first and then classifying (i.e., recognition) of emotion using the extracted features. The significance of individual studies primarily depends on the feature extraction technique, which is the most crucial element in ER. EEG signals are collected through different channels (by placing channel-specific electrodes on the skull). Most studies (mainly the pioneer ones) extract features from the channels individually, and they are collectively used for emotion classification^17,18^. Although setting the electrode position appropriately for any channel is an issue in EEG signal, individually extracted features do not maintain spatial relation (i.e., connectivity) with the other channels. Brain connectivity has become an essential part of the examination in neuroscientific research. Consequently, features related to brain connectivity emerge as an active research focus since they can reflect the relationship between the different brain regions. The effectiveness of brain connectivity features in recognizing emotional states was validated in recent studies^14,19–21^.

Recently, EEG channel connectivity features mimicking the relationship or connectivity between brain regions, represented in a map called a connectivity feature map (CFM), have been widely used in ER^9^. Several methods can measure the relationship between brain regions, e.g., Pearson correlation coefficient (PCC)^20^, cross-correlation (XCOR)^22^, mutual information (MI)^23^, normalized MI (NMI)^24^, phase-locking value (PLV)^25^ and transfer entropy (TE)^14^. XCOR and its variant PCC can detect the linear dependencies, MI and its variant NMI measure the shared information, PLV represents the phase synchronization, and TE measures the directed information flow between two brain regions. The relationship or connectivity between brain areas can be represented as a brain network. The vertices and edges of the network correspond to brain areas and their connections, respectively. If the edges are weighted, they represent the strength of connectivity, then the adjacency matrix (i.e., CFM) is formed, whose elements are the strength of connectivity between different areas. Finally, CFM can be used to classify emotion with a suitable machine learning (ML) or deep learning (DL) method. Many existing EEG-based studies^9,14,19–21^ evaluated on the DEAP benchmark dataset, and ML/DL models were used to classify emotion in Valence and Arousal scales, the emotional measures available along with corresponding EEGs.

This study aims to develop an improved EEG-based ER through innovative information enhancement in CFM formation. Existing ER methods using CFMs considered CFM with a single connectivity method^6,24,26^, and the CFMs are symmetric for undirected connectivity methods where each feature value appears in two places inside a CFM. Thus, the ML or DL methods classify emotion using redundant information when using such CFMs. Classification from redundant information is neither beneficial nor efficient. Alternating a portion of such a symmetric CFM with different informative values might be helpful to enhance the classification performance of ML/DL due to information enhancement. This hypothesis is the main motivation of this study. For information enhancement, CFMs constructed with two individual methods are fused in a single CFM, and the fused CFM holds distinct informative values of the two source CFMs (called base CFMs). Four widely used connectivity methods investigated for base CFM construction are PCC, PLV, MI, and TE, and, the six fused CFMs developed using those base CFMs are PCC + PLV, PCC + MI, PCC + TE, PLV + MI, PLV + TE, and MI + TE. Convolutional neural network (CNN), the prominent DL method, was used for emotion classification in Valence and Arousal scales. Experimental studies also included the ER performance with base CFMs by individual connectivity methods to examine the effectiveness of fused CFM. Rigorous experiments have been conducted on the DEAP benchmark EEG dataset with the constructed CFMs. The major contributions of this study are summarized as follows:

1. Construct base CFMs using PCC, PLV, MI, and TE from EEG, and construct fused CFMs using a pair of individual methods, i.e., PCC + PLV, PCC + MI, PCC + TE, PLV + MI, PLV + TE, and MI + TE.
2. Classify emotion from constructed CFMs using CNN in Valence and Arousal scales.
3. Analyze ER performance among fused CFMs and traditional CFMs, and identify the best-suited CFM fusion for ER.
4. Compare the performance of the proposed ER method with fused CFMs and CNN with existing state-of-the-art methods, and show that the proposed method outperformed those.

The rest of this paper is organized as follows. Section “Related works” describes the related studies of EEG-based ER. Section “EEG-based emotion recognition through information enhancement in CFM” presents the proposed ER method, including data preprocessing, CFM construction, and classification. Section “Experimental studies” presents experimental studies, which include experimental setup, experimental results, and performance comparison. At last, Section “Conclusions” concludes the paper with a few remarks.

## Related works

EEG has been well-studied to investigate how the brain reacts to emotional experiences. Typically, ML or DL methods are used for ER using extracted features from EEG signals. Several ER studies are available using different feature extraction and classification techniques. The features broadly fall under the categories of individual channel features and connectivity features. The following subsections review prominent ER studies categorically based on the EEG features' type.

### ER using individual channel features

Individual channels are considered as independent signal sources in this category, and the characteristic(s) of signal from a particular channel are exposed as feature value(s). Generally, features are extracted from EEG signals in the time domain (e.g., fractal dimension, statistical), frequency domain (e.g., statistical, power spectral density (PSD)), and time–frequency domain (e.g., discrete wavelet transform, Entropy). Pioneer studies considered different ML methods to classify emotions from different time and frequency domain features^17,27^^–^^31^.

Support vector machine (SVM) was used by Liu et al.^17^ to classify discrete emotional states (e.g., happiness, sadness) from PSD features. The PSD features were extracted from six frequency bands for each EEG channel; thus, 6 (band) × $n$ (channel) features were included in each feature set. Apicella et al.^32^ collected EEG signals through an 8‑channel dry electrode cap and classified Valence using neural network (NN) and k-nearest neighbors (KNN). Pane et al.^30^ considered hybrid features of different time domain and frequency domain features to classify emotion using random forest (RF). Jagodnik et al.^28^ extracted different time domain (e.g., mean), frequency domain (i.e., frequency band energies of sub-bands, e.g., Alpha), and nonlinear dynamic (e.g., Entropy) features from EEG; selected features using MI with sequential forward floating selection; finally, classified emotion using SVM, KNN, and RF. Statistical features have been extracted in both time and frequency domains in the study^31^, where 364 features were extracted for each EEG segment, and then feature selection was applied to use the features with the least square SVM and Naive Bayesian (NB) classifier. Subasi et al.^33^ utilized a tunable Q wavelet transform in the feature extraction step and rotation forest ensemble classifier with different classifiers such as KNN, SVM, NN, and RF. Goshvarpour and Goshvarpour^34^ constructed Poincare’s plot (a 2D representation of signal) of EEG signals, extracted features, and classified emotion using SVM, KNN, and NB. In another recent study^35^, they investigated Lemniscate of Bernoulli’s Map (which belongs to the family of chaotic maps) construction from EEG signal and classified emotion using KNN and SVM.

While the aforementioned studies used conventional ML models, DL methods were used in recent studies for emotion analysis, as such methods extract features through their embedded learning process. The differential entropy (DE) feature represented in the 2D map was employed with CNN by Li et al.^18^ to classify three types of emotions (positive, neutral, and negative). Moctezuma et al. used 2D CNN to identify emotions according to Valence and Arousal scales from EEG channels selected by a multi-objective evolutionary algorithm^36^. In the study^37^, a combined CNN + SVM model was used to classify emotions from different time domain features (e.g., where fractal dimension, Hjorth parameters, peak-to-peak, and the root-mean-square) and frequency domain features (e.g., band power, DE, PSD). The study^37^ created feature maps based on topographic (called TOPO-FM) and holographic (called HOLO-FM) representations of EEG signal characteristics. Meanwhile, Li et al.^38^ designed a hybrid model incorporating recurrent NN (RNN) and CNN for emotion classification in the Valence-Arousal plane by using TOPO-FM of the PSDs of the EEG signals. Liu et al.^19^ used statistical characteristics (i.e., variance, mean, kurtosis, and skewness) of the EEG signal as time domain features and used a combined CNN + sparse autoencoder (SAE) + deep NN (DNN) model to classify emotions. The statistical features were extracted from four frequency bands and represented in a 2D map band-wise individually; thus, a 3D map was constructed concatenating features from all four frequency bands. Yuvaraj et al.^39^ also constructed a 3D map staking 2D spatiotemporal representation of EEG signals and then employed a 3D form of CNN for emotion recognition.

CNN‑XGBoost fusion method was applied by Khan et al.^40^ on signal spectrogram images for recognizing three dimensions of emotion, namely Arousal (calm or excitement), Valence (positive or negative feeling), and Dominance (without control or empowered). Moon et al.^21^ used PSD features, which were extracted from ten frequency bands, and SVM and CNN were used as classifiers. ER was performed using simple recurrent units network and ensemble learning by Wei et al.^41^, where the mean absolute value method is employed to extract the time-domain features; the PSD approach is adopted to obtain the characteristics of EEG signals in the frequency domain; and Fractal dimension and DE features were used for nonlinear analysis of the EEG signals. Hurst, sample entropy, Hjorth complexity, vector autoregression, wavelet entropy, spectral entropy, and PSD features were extracted by^42^, where DNN was employed to classify emotions. Song et al.^43^ employed the dynamical graph CNN to classify emotion from five different features, including DE and PSD. Dynamical graph CNN was also used by Asadzadeh et al.^44^, where each emotion was modeled by mapping from scalp sensors to brain sources using a Bernoulli–Laplace-based Bayesian model.

### ER using connectivity feature

EEG connectivity feature is mainly based on connections in brain regions. It is widely accepted that the brain's regions are connected by a network, and the interactions between the network's nodes can be used to interpret brain activity. Thus, emotion analysis seems beneficial in measuring the relationship between several brain areas, and several existing ER studies have revealed the effectiveness of CFM with connectivity features. Existing ER methods using CFMs are mostly considered connectivity features in 2D form. A 2D CFM may be an $n\times n$ feature matrix, where $n$ is the number of EEG channels. Gao et al.^7^ used two connectivity features named Granger Causality (GC) and TE with three classifiers (i.e., SVM, RF, and decision tree) to classify emotional states. GC and TE features were firstly represented in 2D CFMs individually with different sizes, and then applying the histogram of the oriented gradient method, 1D feature vectors were created from the 2D CFMs.

Chen et al.^6^ used three connectivity methods named PCC, PLV, and MI for emotional states classification based on Valence and Arousal scales by SVM. Wang et al.^24^ also used SVM for emotion classification, where Normalized MI (NMI) was used for connectivity feature extraction. Khosrowabadi et al.^45^ used Phase Slope Index, Directed Transfer Function (DTF), and Generalized Partial Direct Coherence for connectivity features and considered KNN and SVM as classifiers. Petrantonakis and Hadjileontiadis^46^ used higher order crossings and XCOR to extract features and SVM as a classifier. Arnau-González et al.^26^ combined the MI feature with spectral power, used a feature selection approach combining Welch’s t-test with principal component analysis (PCA), and classified emotions using NB and SVM.

Many existing studies considered CNN and other DL methods to classify ER from CFMs. Bagherzadeh et al.^47,48^ used PDC and dDTF to extract connectivity features and classify emotions using pre-trained CNN models. Chao et al.^49^ used maximal information coefficient (MIC) for CFM construction, employed a PCA network (PCANet) based DL model for deep feature extraction from constructed CFMs, and used SVM and CNN to classify emotions. Islam et al.^16^ generated 2D CFMs using PCC, developed a different CFM in reduced size by rearranging the values of the CFMs of the upper triangle, and used CNN for emotion classification from both of the CFMs individually. Jin et al.^50^ also used PCC for feature extraction, represented in 1D, and Long Short-Term Memory (LSTM) + NN was employed for emotion classification. Chen et al.^20^ used PCC, PLV, and TE to extract connectivity features and employed domain adaptive residual CNN for emotion classification from CFMs.

A few studies considered PCC with other methods and represented connectivity features in 3D maps. Moon et al.^21^ used PCC, PLV, and PLI to construct CFMs from ten frequency bands and considered SVM and CNN classifiers. The three types of features were represented in a 3D map individually whose size was $n\times n\times 10,$ where $n$ is the number of EEG channels, and 10 is the number of frequency bands. Liu et al.^19^ used PCC for feature extraction from four frequency bands for $n\times n\times 4$ sized CFM, and then they used a combined CNN + SAE + DNN model to classify emotions. The studies^19,21^ revealed that the connectivity features improve the performance over the individual channel feature.

## EEG-based emotion recognition through information enhancement in CFM

It is closely observed from the existing methods that CFM, by a particular connectivity method, is mainly a symmetric 2D matrix having replicate feature values in upper and lower triangles. Such 2D CFM is suitable to place as input of CNN as inherited convolutional operation with the 2D kernel of CNN is its most powerful feature. Therefore, most of the existing studies (such as^14,19–21^) used to train CNN with produced 2D CFM with redundant feature values. According to our knowledge, the study^16^ considered only the upper triangle of 2D CFM that minimizes redundancy, but they reformed the triangle values to 2D matrix form to make it suitable for CNN. Redundant feature values are ineffective in improving the performance of any ML/DL model (e.g., CNN), and more variant but relative information is suitable to improve the model’s performance. As CNN prefers 2D-sized CFM, the information enhancement in 2D CFM is a key issue for better performance by CNN which has been explored and managed through an innovative technique in this study.

**Figure 1:**
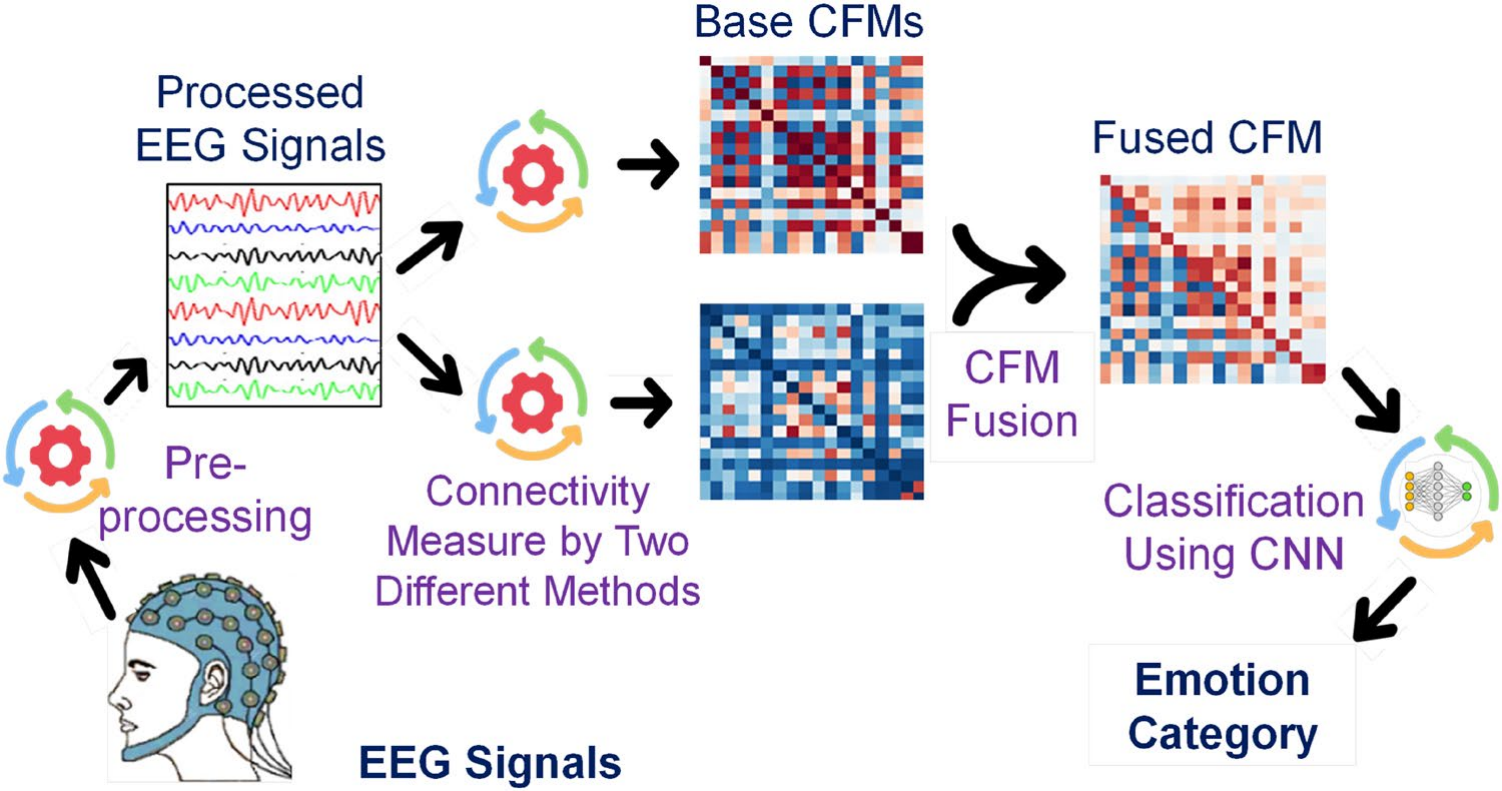
The framework of the proposed emotion recognition system from EEG.

The construction of improved CFM (called fused CFM) for harmonizing relatively more extensive connectivity feature values from EEG signals is the primary issue of the study to develop a well-performed EEG-based ER. CNN is adopted to classify emotion from the fused CFMs. Considering preprocessing of EEG data as a standard step of ER, Fig. 1 demonstrates the proposed ER system. There are four major steps: preprocessing the EEG signals, two CFMs (called base CFMs) construction using two different connectivity methods, fused CFM construction from these two base CFMs, and classification of emotions from the fused CFMs by CNN. The following subsections describe these steps of ER system briefly.

### Benchmark dataset and preprocessing

Database for Emotion Analysis using Physiological Signals (DEAP)^51^, one of the largest EEG datasets for ER, was considered to evaluate the performance of the proposed ER system. It includes EEG and peripheral physiological signals of 32 subjects (16 males and 16 females), where 40 emotional music videos were used as stimuli. Additionally, the database has subjective scores that characterize the emotional states brought on by seeing the movies in terms of their levels of Valence, Arousal, Liking, and Dominance. The DEAP database uses the BioSemi ActiveTwo system to record data. The EEG electrodes are placed according to the 10/20 international standard. This study employed preprocessed EEG signals from the database, which was downsampled to 128 Hz, EOG artifacts were removed, and a band-pass frequency filter with a range of 4.0–45.0 Hz was used. There were 40 channels total, of which 32 were for EEG signals and the remaining channels for peripheral physiological inputs.

In the DEAP dataset, the length of the signal was 63 s: the first 3 s of data were the pre-trial baseline, which was removed, and the last 60 s of data were processed for this study. To increase samples for training, the EEG signals were segmented. An ideal segmentation time window size is 3–12 and 3–10 s, which preserves the key information of Valence and Arousal levels, respectively, as demonstrated by Candra et al.^52^. For this experiment, EEG signals were segmented using an 8 s sliding time window with an overlap of 4 s. Thus, 14 segments were obtained from a 60 s trial, and the total segments for 32 participants were 14 × 32 (participants) × 40 (trial) = 17,920; those are the samples to construct CFM. It is reported that emotion is highly related to Gamma and Beta sub-bands^16^. Only the Gamma sub-band was considered in this study for final evaluation. EEGLAB^53^, an open-source toolbox, was used to extract sub-bands from the EEG signal. Among the four quality levels available in the dataset, Valence and Arousal are the two well-studied scales which were chosen for ER in this study. In the dataset, each of the Valence and Arousal values ranges from 1 (low) to 9 (high). The scales were divided into two parts for ER task as binary classification. Similar to the work in^16^, Valence or Arousal is considered as high for values above 4.5 and low for less than or equal to 4.5. At a glance, Valence and Arousal classifications must be performed through two independent binary classification tasks. By combining Valence and Arousal, human emotions (e.g., Angry, Happy, Sad) can be expressed; often, these are visualized using Russell’s circumplex model of emotions^54^.

### Connectivity feature map (CFM) construction and fusing CFM

The feature extraction technique transforms inputs to new dimensions, which are different (linear, nonlinear, directed, etc.) combinations of the inputs. The strength of connectivity between two electrodes reflects an interaction between two cortical areas during one experiment. This interaction could be a direct correlation or inverse correlation, synchronization, or asynchronization, depending on what aspects are investigated. Relationships vary depending on the connectivity types as well. Four diverse connectivity methods are chosen for this study: PCC, PLV, MI, and TE. Among the selected methods, PCC is a linear functional connectivity method, PLV and MI are nonlinear functional connectivity methods, and TE is a nonlinear effective connectivity method. Following subsections briefly describe CFM construction using the four methods and improved CFM construction fusing base CFMs.

#### CFM construction using individual methods

Pearson correlation coefficient (PCC) measures the linear correlation between two signals $X$ and $Y$, which can be calculated as

1 $${PCC}_{XY}=\frac{{\varvec{n}}\sum {{\varvec{X}}}_{{\varvec{i}}}{{\varvec{Y}}}_{{\varvec{i}}}-\sum {{\varvec{X}}}_{{\varvec{i}}}\sum {{\varvec{Y}}}_{{\varvec{i}}}}{\sqrt{{\varvec{n}}\sum {{\varvec{X}}}_{{\varvec{i}}}^{2}-{\left(\sum {{\varvec{X}}}_{{\varvec{i}}}\right)}^{2}}\sqrt{{\varvec{n}}\sum {{\varvec{Y}}}_{{\varvec{i}}}^{2}-{\left(\sum {{\varvec{Y}}}_{{\varvec{i}}}\right)}^{2}}},$$

where $n$ is the sample size, ${X}_{i}$, ${Y}_{i}$ are the individual sample points indexed with $i$. The value of PCC ranges from − 1 to 1. (− 1): complete linear inverse correlation between the two signals, (0): no linear interdependence, (1): complete linear direct correlation between the two signals.

Phase-locking value (PLV) describes the phase synchronization between two signals, which is calculated by averaging the absolute phase differences as follows-

2 $$PLV\left(X,Y\right)=\frac{1}{T}\left|\sum_{t=1}^{T}\mathit{exp}\left\{j({\varphi }_{X}^{t}-{\varphi }_{Y}^{t})\right\}\right|.$$

Here, ${\varphi }^{t}$ is the phase of the signal at time t, $X$ and $Y$ denote two electrodes, $T$ is the time length of the signal. The value of PLV ranges from 0 to 1, indicating that the two signals are either perfectly independent or perfectly synchronized, respectively.

Mutual Information (MI) is an information theoretic approach to measuring shared information between two variables. The amount of information about one random variable that may be learned from observing another is measured as MI. The following is the definition of MI between two random variables, $X$ and $Y$:

3 $$MI\left(X, Y\right)=H\left(X\right)+H\left(Y\right)- H\left(X,Y\right)$$

In this case, H stands for Shannon entropy^55^. For calculating the probability that is required to calculate Entropy, the fixed bin histogram approach was followed. The number of bins selected for all the calculations is 10. The marginal entropies of the two variables $X$ and $Y$ are $H\left(X\right)$ and $H\left(Y\right)$, respectively, and their combined Entropy is $H\left(X,Y\right)$. MI is symmetric and nonnegative. The range of MI's value is: $0\le MI(X,Y)<\infty$. If $MI\left(X, Y\right)$ is equal to 0, then $X$ and $Y$ are independent. If $MI\left(X, Y\right)$ is greater than 0, then $X$ and $Y$ are dependent.

The transfer entropy (TE) measures the directed flow of information from a time series or signal $Y$ to another signal $X$. In other words, it describes the gain obtained by knowing $Y$ for the prediction of $X$.

4 $${TE}_{Y\to X}=H\left({X}_{t},{Y}_{t}\right)-H\left({X}_{t+h},{X}_{t},{Y}_{t}\right)+H\left({X}_{t+h},{Y}_{t}\right)-H\left({X}_{t}\right)$$

If $w$ is the future of $X,$ i.e.,${X}_{t+h}$, transfer Entropy ${T}_{Y\to X}$ can be computed as a combination of entropies:

5 $$TE(w, X,Y)=H(w,X,)+H(X,Y) - H(X) - H(w,X,Y)$$

The ranges of TE value are $0{\le TE}_{Y\to X}<\infty$. If the TE value is equal to 0, then there is no causal relationship between the time series. TE value greater than 0 indicates that a causal relationship exists between them.

**Figure 2:**
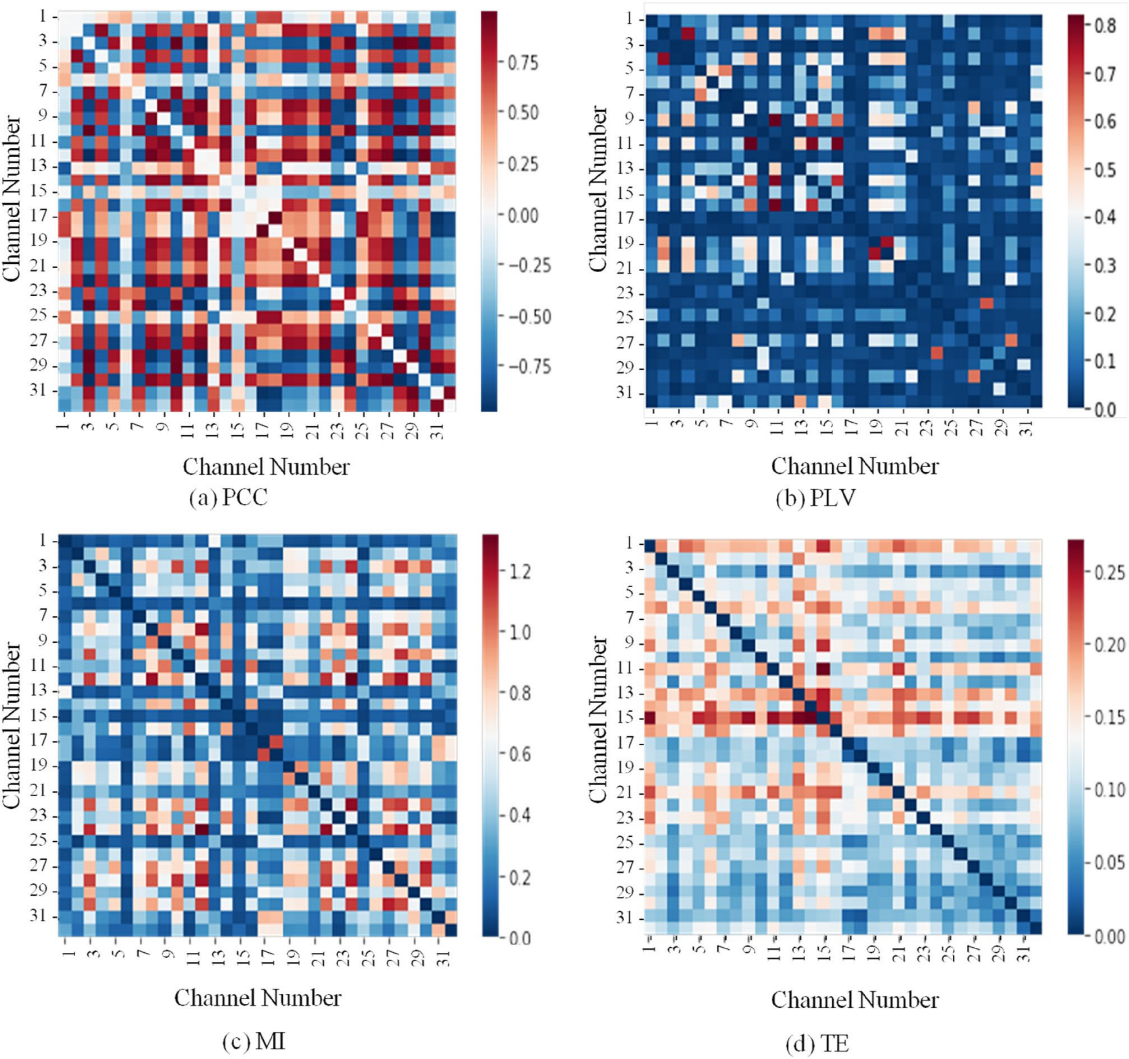
Heatmap representation of sample connectivity feature maps (CFMs) constructed with individual methods. Seaborn library of Python (https://seaborn.pydata.org/) is used to generate heatmap.

In the case of CFM, the variables are signals from individual EEG channels. The connectivity features were calculated for every pair $(X,Y)$ of EEG channels. Therefore, if there are $N$ channels, the number of obtained features is $N(N-1)/2$ for undirected connectivity (e.g., PCC) and $N(N-1)$ for directed connectivity (e.g., TE). The connectivity features for all channel pairs can be represented in a matrix, and Fig. 2 shows the heatmap representation of sample CFMs constructed with individual methods. The element of the matrix at the position $\left(X,Y\right)$ indicates the connectivity between the EEG signals obtained from the $X$ th and $Y$ th channels. The values of location $(X,X)$ or $(Y,Y)$ were set to zero, as these are not information between two different channels. If there are $N$ channels, then every feature map has $N$ rows and $N$ columns. As there are 32 channels in the DEAP dataset, thus every feature map has 32 rows and 32 columns. Figure 2a shows a sample CFM constructed with PCC, which indicates the correlation between signals collected from two EEG channels. More specifically (as an example), the higher value of the matrix at position (2, 4) indicates that the signals collected from channel 2 and channel 4 are highly correlated, while the lower value of the matrix at position (2, 3) indicates that the signals collected from channel 2 and channel 3 are inversely correlated. Similarly, phase synchronization, dependency, and causal relationship between two signals are indicated by Fig. 2b,c,d respectively. It is observed from Fig. 2 that the elements of a matrix at position $\left(X,Y\right)$ and $\left(Y,X\right)$ are the same (i.e., CFMs are symmetric) for functional connectivity methods PCC, PLV, and MI. However, these are not the same (i.e., asymmetric CFM) for effective connectivity method TE. Another important observation from the figure is that value ranges are different in different CFMs due to the inherited properties of individual connectivity methods. Among the four individual methods, CFM using PCC holds a large variation in their values, and it is -0.99 to 0.98 in the sample presented in Fig. 2a. The values in CFMs by PLV and MI vary from 0.0 to 0.82 (for PLV) or more than 1(for MI). At the same time, the values for TE vary from 0.0 to 0.27.

#### Improved CFM construction fusing base CFMs

**Figure 3:**
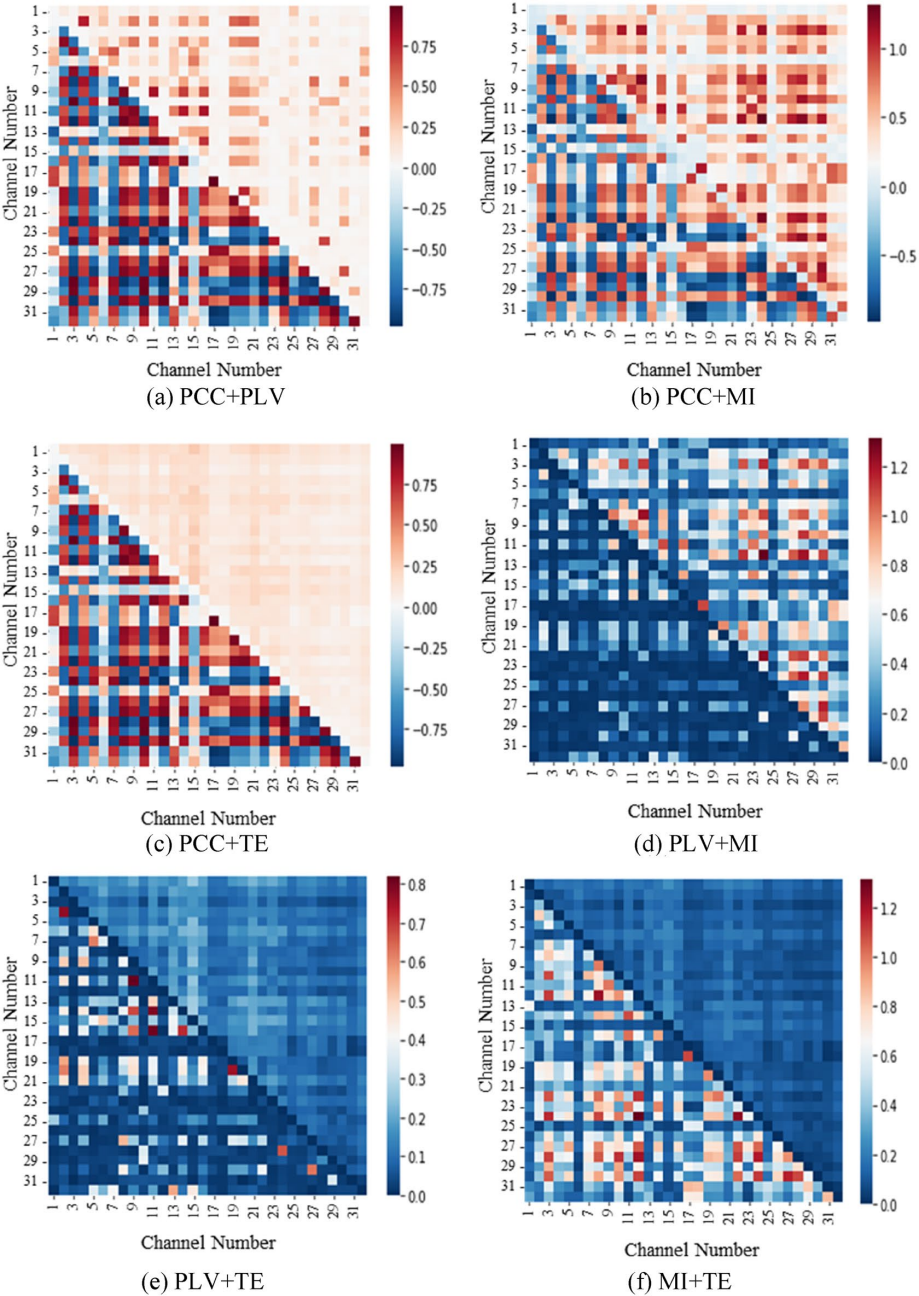
Heatmap representation of sample fused connectivity feature maps (CFMs) constructed with every two different methods. Seaborn library of Python (https://seaborn.pydata.org/) is used to generate heatmap.

Improved CFM Construction fusing CFMs constructed by individual methods is the central significance of the present study. Six new CFMs can be constructed by combining (i.e., fusing) every two of the four individual CFMs depicted in Fig. 3 as heatmaps. As an example, Fig. 3a shows a sample CFM constructed with PCC + PLV where the upper triangular portion (elements of the matrix at position $\left(X,Y\right)$ for $X<Y$) contain features extracted with PLV, and the lower triangular (for $X>Y$) portion holds PCC features. Similarly, CFMs constructed with PCC + MI, PCC + TE, PLV + MI, PLV + TE, and MI + TE are shown in Fig. 3b,c,d,e,f, respectively. It is already discussed that CFM constructed with TE is asymmetric; therefore, it is a matter of choice to select between upper triangular or lower triangular to fuse with the other three methods (i.e., PCC, PLV, and MI). Since the variation between upper and lower triangular TE CFM is not much, only one is considered to combine with the other and is shown in Fig. 3.

The information enhancement without enlarging size is the main significance in CFM fusion in Fig. 3 over the CFMs with individual methods of Fig. 2. The CFMs in Fig. 2 hold redundant information completely or partially. CFMs with PCC, PLV, and MI are symmetric and provide complete redundancy of individual values, whereas CFM with TE also holds similar values in upper and lower portions and hold partial redundancy. While redundant information only increases the computation burden in ML without any benefit in decision making, fusing CFM is beneficial for ML as its upper and lower portions were managed from two different connectivity methods and enhanced information in CFM. While the sizes of fused CFMs (in Fig. 3) are the same as those of individual methods (in Fig. 2), fusing CFM is a cost-effective as well as efficient information enhancement for ML.

Differences in value ranges (i.e., the difference between the highest and the lowest range) in CFMs by individual methods (e.g., PCC, PLV) expose diversity in CFMs constructed by combing individual methods. It is already observed from CFMs of individual methods (in Fig. 2) that values for PCC hold large variations, then PLV and MI, and variation for TE is the lowest. Therefore, when PCC combines with another method, resulted CFM shows a large variation in the value ranges. Among the six fused CFMs, PCC + MI shows the highest values variability, and PLV + TE shows the lowest variability as the CFMs presented in Fig. 3. It is notable that the CFM figures are colorized with relative values (i.e., the lowest one is blue, and the highest one is red) the colors for values for individual methods (in Fig. 2) are changed in the combing cases (in Fig. 3). Finally, CFMs with combing two individual methods enhanced the data value variation at a glance, which is also an element to enhance the performance of ML.

### Emotion classification using convolutional neural network (CNN)

Among different DL methods, CNN is the most successful classifier for two-dimensional (2D) data and can implicitly extract relevant features^56,57^. Since the constructed CFMs are in 2D, CNN was chosen as a suitable classifier. In general, a CNN architecture consists of an input layer, several convolutional-subsampling layers, a flatten layer, a fully connected layer, and an output layer. The first operation of a CNN is convolution performed on the input (matrix, image, or map) with its kernel, which generates a new convolved matrix. Preceding subsampling operation will downsize the convolved matrix with important features. After one or more convolutional-subsampling operations through a fully connected dense layer, the output layer categorizes the given 2D matrix as input of the CNN. The general description of CNN and its operations are available in existing studies, where CNN and its architectural issues are the primary concern^56,58^.

**Figure 4:**
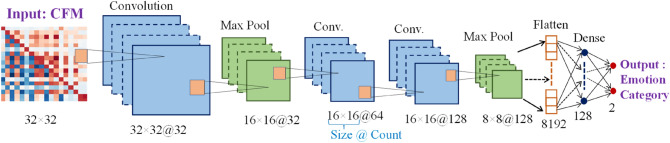
CNN architecture (with dimension of individual layers) to classify emotions from CFMs.

Figure 4 shows the CNN architecture to classify emotions from 2D CFMs; such architecture has been used in recent EEG-based studies^9^. Three convolutional layers, two max-pooling layers, flatten layer, a dense layer, and an output layer make up the CNN architecture employed in this study. In the figure, the size of generated 2D shape and the number of shapes are marked for each convolutional and pooling layer. Every convolution layer used kernels of size 3 × 3, and the stride was set to 1. Rectified linear unit (ReLU) was used as an activation function. The numbers of filters were 32, 64, and 128 for the 1st, 2nd, and 3rd convolution layers, respectively. The same convolution (padding = 1) is used for all the convolution layers to preserve the information from the pixels of a corner of the input feature map. Two max-pooling layers are used, one is after the first convolution layer, and another is after the third convolution layer. The 2 × 2 sized kernels with stride 2 were used in every pooling layer. After each max-poling layer, batch normalization was used to accelerate the model training. After convolution and pooling operations, the feature maps were flattened to a single-column vector of 8192 (= 8 × 8 × 128) and fed to the dense layer. The dense layer and output layer's respective neuron counts were set at 128 and 2, respectively, and the dense layer is accompanied by a 25% dropout. In the output layer, the "Sigmoid" activation function was applied. Table 1 shows the shape and hyperparameter of the CNN model.

**Table 1:** Shape and hyperparameter of CNN model.

Layer and type	Input shape	Kernel size, count	Pooling size	Stride	Output shape
Conv2D	32 × 32 (CFM)	3 × 3, 32	–	1 × 1	32 × 32 @ 32
MaxPooling2D	32 × 32 @ 32	–	2 × 2	2 × 2	16 × 16 @ 32
Conv2D	16 × 16 @ 32	3 × 3, 64	–	1 × 1	16 × 16 @ 64
Conv2D	16 × 16 @ 64	3 × 3, 128	–	1 × 1	16 × 16 @ 128
MaxPooling2D	16 × 16 @ 128	–	2 × 2	2 × 2	8 × 8 @ 128
Flatten	8 × 8 @ 128	–	–	–	8192
Dense (Hidden)	8192	–	–	–	128
Dense (Output)	128	–	–	–	2 (Emotion Category)

## Experimental studies

This section presents experimental results and analyses of ER systems with CFMs created by different methods (i.e., MI, NMI, and PMI) individually and fused CFMs on the DEAP EEG dataset. The efficacy of the method was assessed based on the test set recognition accuracies. Finally, the outcomes of the study were compared with the state-of-the-art methods. However, the experimental setup and evaluation metric are described briefly first.

### Experimental setup and evaluation metric

Keras and TensorFlow frameworks of Python were used for implementing the CNN models. The CNN was trained by the Adam algorithm^59^, and binary cross-entropy was used as the loss function. The learning rate, batch size, and epochs for the CNN were set to 0.00001, 32, and 500, respectively. A fivefold cross validation (CV) was applied where 20% of the available data were reserved as a test set by turn, while 80% of the data was used to train the model. Moreover, the performance was also evaluated for fixed training and test sets in several cases. The P100 GPU in the Kaggle platform was used for training the model, and MATLAB R2021a was used for feature extraction through the device of configuration: CPU: Intel(R) Core(TM) i5-4200 CPU @ 2.50 GHz, RAM: 4.00 GB, 64-bit windows operating system.

The performance of the implemented model was evaluated using the three most widely used evaluation metrics (i.e., sensitivity, specificity, and accuracy), which can be expressed as:

6 $$Specificity = TN/(TN+FP)$$

7 $$Sensitivity = TP/(TP+FN)$$

8 $$Accuracy= (TP+TN)/(TP+TN+FP+FN)$$

Here TP or true positive means the samples were originally labeled as high, and the model also predicted those as high, TN or true negative means the samples were originally labeled as low, and the model also predicted those as low, FP or false positive means the samples were originally labeled as low, but the model predicts those as high, FN or false negative means the samples were originally labeled as high but the model predicts those as low. Sensitivity is the percentage of true detected high-labeled samples to total high-labeled samples, and specificity is the percentage of true detected low-labeled samples to total low-labeled samples. An excellent classifier should have high sensitivity and specificity at the same time. Notably, performance on the test set is more desirable, representing the generalization ability of an ML/DL system.

### Experimental results and analyses

**Figure 5:**
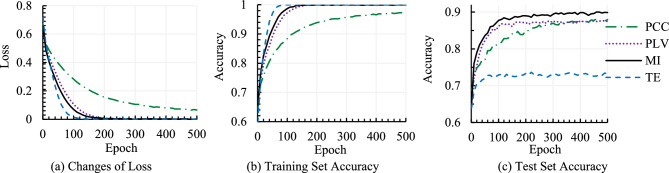
Model loss and accuracy for Valence classification using CFMs with individual connectivity method.

Model’s loss and accuracy curves for Valence and Arousal classification for a sample run are analyzed first, and then the classification results are presented for both fivefold CV and training-test split mode. The performance of different sub-bands (i.e., Alpha, Beta, and Gamma) and full frequency band have been evaluated with individual connectivity feature maps (CFMs) for a more reasonable comparison. Since the notion of training loss and accuracy are found to be similar for all the sub-bands, graphical illustrations of varying training epochs are presented for the Gamma band only in Fig. 5. Figure 5 shows the model’s training loss and accuracy curves on both training and test sets for Valence classification for a sample run where CFMs are constructed using the individual connectivity method. In ML, the performance of the training set indicates the learning or the memorization of the patterns used in training a model. At the same time, performance on the test set indicates the generalization ability (i.e., performance well behind the training data) of a model. According to Fig. 5a, the loss convergence for TE is faster than any other method, and similarly, the accuracy improvement in Fig. 5b. In the test set’s accuracy, TE shows the worst performance, whereas MI achieved the highest accuracy, as seen in Fig. 5c. The test set accuracies of PCC and PLV are competitive. The test set accuracy is inferior to the training set score for the model. The scenario is acceptable because the test set was reserved for checking the performance of unseen data (i.e., not used in the training process), and lower performance on the test set than the training set is common in the ML domain. Test set performance (i.e., generalization ability) is the key performance measure element of a model and is used to compare with other related models.

**Figure 6:**
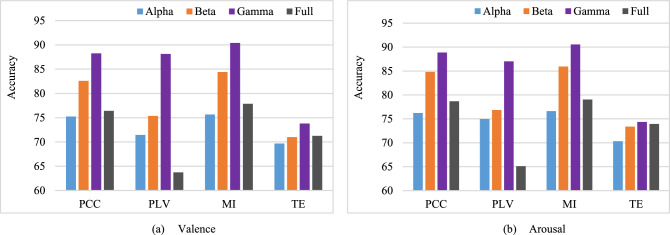
Valence and Arousal classification accuracies with individual connectivity methods in different frequency sub-bands and full frequency band.

Figure 6 compares test set classification accuracies with CFMs by the individual connectivity methods for Alpha, Beta, Gamma and full frequency bands. As mentioned earlier, it is reported in the literature that the sub-bands may yield more accurate information about constituent neuronal activities^15^, and emotion is highly related to the Beta and Gamma sub-bands than the Alpha sub-band. The experimental results presented in Fig. 6 also justify the matter of frequency band compatibility for emotion recognition. According to the figure, the recognition accuracy is generally higher in sub-bands than full EEG frequency band for any CFM construction method. Again, accuracies for the Gamma sub-band are better than the Alpha and Beta sub-bands for both Valence and Arousal classifications. Recent studies also demonstrated such observation^9^. Therefore, owing to achieving better accuracy, further experimental outcomes have been observed for the Gamma band only for simplicity in keeping the paper concise.

**Figure 7:**
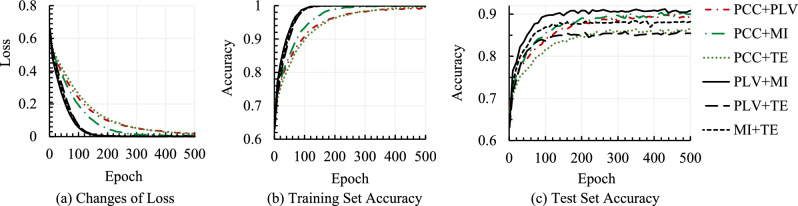
Model loss and accuracy for Valence classification using fused CFMs with two connectivity methods.

Figure 7 shows the model’s loss and accuracy curves on both training and test sets for Valence classification for a sample run with fused CFMs where CFMs are constructed using a combination of every two connectivity methods. As the two CFMs constructed with two angular portions of TE showed similar characteristics, and one of them is displayed here. According to Fig. 7a, the loss convergence for the methods where PCC is combined with any other methods (i.e., PCC + PLV, PCC + MI, PCC + TE) is slower than any other methods, and similarly, the accuracy improvement in Fig. 7b. TE combined methods show lower test sets’ accuracy where PLV + MI achieved the highest accuracy, as seen in Fig. 7c. The accuracies of PCC + MI and PCC + PLV are competitive.

**Figure 8:**
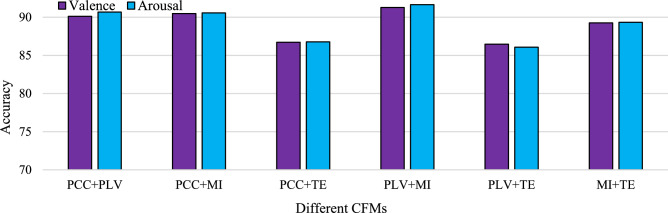
Test set accuracies for CFM with individual methods and fused CFMs in Valence and Arousal classification for Gamma sub-band.

Interestingly, test set accuracy with MI is better than others, as found in Fig. 5c, and the MI combined method PLV + MI is better than others, as found in Fig. 7c. Similar characteristics can also be observed for TE, which obtained the lowest test set accuracy in Fig. 5c, and the TE combined method PLV + TE obtained the lowest test set accuracy among the combined methods, as found in Fig. 7c. At a glance, PLV + MI achieved better accuracies than any other individual or combined CFM method. Figure 8 summarizes the test set accuracies in the training-test split mode for six fused CFMs in Valence and Arousal scales. According to the achieved classification accuracies presented in the figure, the PLV + MI method achieves the highest Valence and Arousal classification accuracies, which are 91.29% and 91.66%, respectively. On the other hand, the worst achieved accuracy was with PLV + TE.

Table 2 demonstrates test set classification performance in different evaluation metrics for Valence and Arousal with different CFMs for fivefold CV and training-test split modes. The training-test split may consider as one individual case among the five cases of fivefold CV mode, and similar performance is also observed among the different CFMs cases. The best performance for a particular evaluation metric and mode is placed in boldface. As an example, for Valence and Arousal classification, MI achieved the best specificity, sensitivity, and accuracy in both modes. The lowest results were achieved with the TE feature in both modes. The performances of PCC and PLV are competitive. At a glance, MI shows superior performance over other connectivity methods.

**Table 2:** Classification comparison using different individual CFM methods.

		Fivefold CV				Training-test sets split as 80–20%			
		PCC	PLV	MI	TE	PCC	PLV	MI	TE
Valence	Specificity (%)	82.26	81.37	**85.27**	55.28	83.28	79.42	**85.91**	46.10
	Sensitivity (%)	90.45	91.31	**92.74**	83.70	90.80	92.97	**93.01**	89.96
	Accuracy (%)	87.43	87.64	**89.98**	73.22	88.03	87.97	**90.40**	73.80
Arousal	Specificity (%)	81.04	81.63	**84.06**	54.66	83.07	83.07	**85.68**	44.41
	Sensitivity (%)	92.30	91.97	**93.60**	84.45	92.60	92.38	**93.30**	91.37
	Accuracy (%)	88.22	88.22	**90.14**	73.65	89.15	89.01	**90.54**	74.36

Significant values are in bold.

Table 3 presents the classification comparison using different combined connectivity feature map methods. From the table, it can be observed that PCC + PLV has achieved the highest specificity for Valence classification in fivefold CV mode. For Arousal classification, PCC + MI has achieved the highest specificity in the fivefold CV mode. PLV + MI has obtained the highest specificity for both Valence and Arousal classification in training-test split mode. In the case of sensitivity and accuracy, PLV + MI has shown the best performance for both Valence and Arousal classification in both fivefold CV and training-test split modes.

**Table 3:** Classification comparison using different fused CFM methods.

		Five(5)-fold CV						Training-test sets split as 80–20%					
		PCC + PLV	PCC + MI	PCC + TE	PLV + MI	PLV + TE	MI + TE	PCC + PLV	PCC + MI	PCC + TE	PLV + MI	PLV + TE	MI + TE
Valence	Specificity (%)	**85.32**	84.32	79.01	85.27	79.05	83.39	84.19	85.01	77.45	**86.00**	79.03	83.20
	Sensitivity (%)	92.75	92.85	90.33	**93.89**	89.38	91.94	93.58	93.68	92.13	**94.38**	90.80	92.79
	Accuracy (%)	90.01	89.71	86.16	**90.71**	85.57	88.79	90.12	90.49	86.72	**91.29**	86.47	89.26
Arousal	Specificity (%)	84.80	**85.16**	76.84	85.12	78.01	81.26	84.60	85.84	77.75	**86.15**	79.13	82.21
	Sensitivity (%)	92.97	93.42	91.88	**94.29**	90.30	93.21	94.13	93.25	91.90	**94.78**	90.02	93.39
	Accuracy (%)	90.01	90.43	86.43	**91.15**	85.84	88.88	90.68	90.57	86.77	**91.66**	86.08	89.34

Significant values are in [bold].

**Figure 9:**
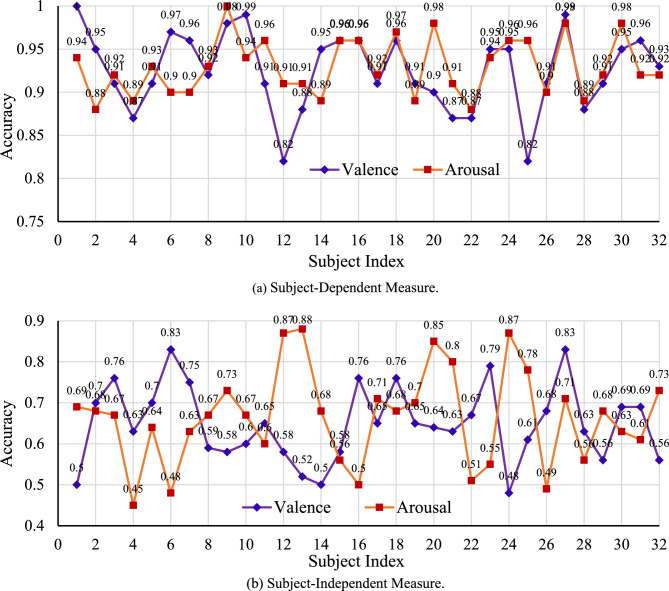
Subject-dependent and subject-independent test set classification accuracies with fused CFM with PLV + MI.

While the proposed method has been found effective in the experiments conducted by shuffling samples for all 32 subjects, it is also interesting to know how it performs individual subject basis (i.e., subject-dependent) and cross-subject basis (i.e., subject-independent). Different experiments have been conducted to evaluate the proposed method for subject-dependent and subject-independent issues. In subject-dependent cases, for a particular subject, the available 560 samples (= 14 segments × 40 trials) were shuffled, 448 samples (i.e., 80%) were used to train the CNN, and the rest 112 samples (i.e., 20%) have been reserved as the test set. Figure 9a shows Valance and Arousal test set classification accuracies for 32 subjects individually for fused CFM by PLV + MI as it has outperformed others. For Valance classification, 100% accuracy (i.e., truly classified all 112 test samples) has been observed for Subject 1 only, and the worst accuracy has been found to be 82% for Subject 12 and Subject 25. In the case of Arousal classification, the method showed 100% accuracy for Subject 9 only, and the lowest accuracy was 87% for Subject 22. The subject-dependent average accuracy for both Valance and Arousal cases is around 93%, better than all subjects shuffled together. Subject-dependent better performance is logical as samples were used to train the CNN for the same subject. At the same time, such performance justified the proficiency of the proposed method for emotion classification in individual subject cases.

Figure 9b shows Valance and Arousal classification accuracies for fused CFM by PLV + MI in the subject-independent measure through leave-one-subject-out mode, where classification accuracies were measured for a particular subject (with 560 samples). At the same time, CNN training was performed with 17,360 (= 560 × 31) samples of the remaining 31 subjects. The Valance and Arousal accuracies for subject-independent cases are generally inferior to the subject-dependent case. However, higher than 80% accuracies for Valance classification in two subjects (i.e., 6 and 27) and Arousal classification in four subjects (i.e., 12, 13, 20, and 24) are promising outcomes. In addition, five other subjects (i.e., 3, 7, 16, 18, and 23) for Valance and six other subjects (i.e., 9, 17, 21, 25, 27, and 32) for Arousal are shown accuracy higher than 70%. Better performance on several subject-independent cases is achievable when similar patterns are available in training samples. On the other hand, when the test subject samples are largely different from the training subjects’ samples, it is common to get inferior accuracy. The inferior outcomes (say, accuracy below 70%) for a number of subject cases indicate that the corresponding subjects’ samples are largely dissimilar from other subjects. At a glance, the average subject-independent classification accuracies are around 65% for both Valance and Arousal cases. The achieved subject-independent classification accuracy is better than or competitive with the reported accuracies in several studies such as^28,60^. However, it is a remaining challenging issue for the proposed method to achieve better subject-independent classification accuracy by analyzing CFM construction and employing different DL models.

**Figure 10:**
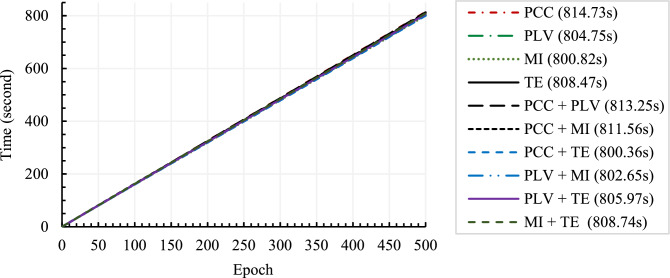
CNN training time for the models with different CFMs.

Figure 10 presents the time required to train the CNN with four based CFMs (of four individual connectivity methods) and six fused CFMs. The legend of the figure indicates the required time to train with the name of the respective CFM. It can be observed from the figure that the time needed to train with different CFMs is almost the same (and they together seem like a single bold line). For example, PCC requires 814.73 s, and PCC + PLV requires 813.25 s to train the model up to 500 epochs. The figure revealed that using fused CFMs does not incur additional computational costs. The reason is apparent because the size of a fused CFM is the same as that of individual base CFMs, and the CFM size is always 32 × 32 matrix, as explained in Section “EEG-based emotion recognition through information enhancement in CFM”. For the same 32 × 32 sized input CFM and the same CNN architecture, CNN training times are expected for the base and fused CFMs to be unchanged. However, CFM fusion may look like an additional task in the proposed method over the training CFM-based methods, but the CFM fusing task is computationally negligible as fusion only replaces a portion of CFM with another CFM. Finally, the CFM fusion takes place before the training of CNN, and therefore, CNN training time remains the same.

### Performance comparison with existing methods

Table 4 compares the Valence and Arousal test set classification accuracies obtained in this study with other connectivity feature-based ER studies on the DEAP dataset. The existing methods are diverse in segmentation time and overlapping, classifier consideration, and validation methods (i.e., the mode of test sample reservation: fixed training—test sample split ratio or cross-validation). Due to such variations, unbiased fair performance comparison based on a particular element is ponderous. However, classification accuracy variation with segmentation time and overlapping are more visible than other elements (e.g., classifier uses, validation method). Segmentation splits an original EEG sample into several individual samples depending on the time window and overlapping, and a larger overlapping produces more samples (i.e., CFM). With 3 s segment window with 0 s overlap (i.e., no overlap), there are 20 samples for 60 s EEG signal in the study^16^; whereas, with 2.5 s overlap (= 2.5/3 = 83%), there are 100 + samples for the same 3 s segment window in the study^21^. For PCC, the best accuracy was achieved by the study^21^ having larger overlapping than others; the achieved Valance classification accuracy is 94.94% by CNN in fivefold CV mode. The study^19^ considered two segmentation time windows, 8 s and 12 s, with different overlapping 50% and 66%, using CNN to classify emotion. The segmentation time window of the study is longer than other studies (e.g.,^16,21^), and it achieved accuracies higher than the study^16^ but lower than the study^21^. The study^19^ stated that with higher overlapping, the neighboring data segments become more similar, i.e., features could be learned better when similar information is included in each trial. The study^19^ also shows that a longer segmentation time window reduces accuracy. As a shorter segmentation produces large samples, it is beneficial to train an ML/DL model properly and give better performance. On the other hand, a single CFM was produced from an original EGG sample for no segmentation cases in studies^6,24,26^, and achieved performance was always worse regardless of the classifiers used and the validation methods considered. However, when the overlap is too high, the number of data segments is larger, which requires a longer time for the training time of the ML/DL model.

**Table 4:** Performance comparison of the proposed method with other connectivity feature studies on DEAP dataset.

CFM method	Work References	CFM size	Segmentation time window (overlapping)	Classifier	Train-test split/cross validation	Test set accuracy (%)	
						Valence	Arousal
PCC	^6^	32 × 32	No segmentation	SVM	Leave 01 trial out	72.91	72.34
	^16^	23 × 23	3 s (0 s)	CNN	90%–5%–5% (Sep. validation set)	78.22	74.92
	^21^	32 × 32 × 10	3 s (2.5 s)	CNN	fivefold CV	94.44	–
	^19^	32 × 32 × 4	8 s (4 s)	CNN + SAE + DNN	80–20%	89.49	92.86
			12 s (8 s)	CNN		75.13	76.12
			8 s (4 s)			78.80	82.25
PLV	^6^	32 × 32	No segmentation	SVM	Leave 01 trial out	73.75	71.88
	^21^	32 × 32 × 10	3 s (2.5 s)	CNN	fivefold CV	99.72	–
	^20^	32 × 32	3 s (2.5 s)	DARCNN	tenfold CV	95.15	94.84
					Leave 01 subject out	88.28	87.60
MI	^6^	32 × 32	No segmentation	SVM	Leave 01 trial out	76.17	73.59
	^26^	32 × 32	No segmentation	SVM		60.10	63.50
NMI	^24^	32 × 32	No segmentation	SVM	80–20%	75.16	74.41
TE	^20^	32 × 32	3 s (2.5 s)	DARCNN	tenfold CV	89.06	89.73
					Leave 01 subject out	81.50	81.39
PLV + MI (Present study)		32 × 32	8 s (4 s)	CNN	80–20%	91.29	91.66
					fivefold CV	90.71	91.15

In this study, CFM samples have been produced with a length of 8 s segment with 4 s overlap, and the best test set accuracies by CNN have been achieved with the fused CFM with PLV + MI. On reserved 20% of test samples, the achieved accuracies are 91.29% for Valence and 91.66% for Arousal. Moreover, the accuracies in fivefold CV cases are 90.71% and 91.15% for Valence and Arousal, respectively. A fivefold CV may consider the average of five independent runs of 80–20% training-test split modes; therefore, performance variation is usual. The achieved performance of this study is better than the traditional ML-based studies of ^6,24,26^, regardless of the validation modes considered. While compared to the recent DL studies, the proposed method is better than^19^ and is competitive with^20,32^. In terms of segmentation, DL model, and validation mode, the present study is close to^19^; the study achieved accuracies for 8 s of segmentation by CNN are 78.80% and 82.25% for Valance and Arousal, respectively; the performance is inferior to our method even it considered high dimensional 3D CFM having size 32 × 32 × 4. On the other hand^20,32^ studies considered 3 s segmentation with 2.5 s overlap; thus, better performance over the proposed approach is justified. Moreover, the study^20^ considered a hybrid DL model and different validation modes; the study^21^ considered high-dimensional 3D CFM with and size of 32 × 32 × 10. Such complex DL model and heavy CFM might also be the reasons to get better performance with CFM by PLV.

It is observed from Table 4 that the existing methods are roughly in three categories based on CFM size and shape consideration: 32 × 32 sized 2D CFM, 3D CFM in different sizes and shapes, and 23 × 23 sized 2D CFM. As the DEAP dataset holds EEG signals of 32 channels, 32 × 32 sized 2D CFM construction is the basic approach by individual connectivity methods; several existing studies (i.e.,^6,20,24,26^) and the proposed method used of this study considered 32 × 32 sized 2D. Despite the same 32 × 32 size, CFM values of the proposed method are significantly different with respect to the existing ones. Existing studies generally produced symmetric CFMs using a particular connectivity method (e.g., PCC, PLV, MI) that holds redundant connectivity values in upper and lower triangles in the CFM. On the other hand, the proposed method considered fused CFM where upper and lower triangles hold connectivity values from two different base CFMs produced by two individual methods, e.g., PLV and MI. Regardless of the segmentation and validation mode, the proposed method with 32 × 32 sized 2D CFM outperformed the methods of^6,24,26^ and justified the effectiveness of the fused CFM consideration. The better performance by PLV in^20^ is also acceptable due to shorter segmentation and hybrid DL model consideration, as already discussed. Similarly, high dimensional 32 × 32 × 10 sized 3D CFMs of PCC and PLV with 3 s segmentation in the study^21^ are shown better performance than the proposed method.

The proposed method is close to the study regarding redundancy minimization of CFM values, but the proposed method seems more practical. Among the existing studies, only one study^16^ considered smaller 2D CFM. The 23 × 23-sized CFM is the reformed version of the upper triangle of 32 × 32-sized symmetric 2D CFM produced by PCC. Using an upper triangle minimizes the redundancy issue of the base CFM by PCC. Nevertheless, the performance by CNN on 5% of test samples, while trained by 90% (similar to tenfold CV), is not remarkable; the method achieved test set accuracies of 78.22% and 74.92% for Valance and Arousal classification, respectively. The proposed fused CFM of this study also minimizes redundancy, and at the same time, it also enhances information, including connectivity values from a different CFM. The test set accuracies of the proposed method with fused CFM with PCC (i.e., PCC + PLV, PCC + MI, PCC + TE) are higher or around 90% for both Valance and Arousal cases, as reported in Table 3 of Section “Experimental results and analyses”. Such remarkable outperformance revealed the effectiveness of fused CFM consideration and identified the proposed method as a best-suited promising EEG-based ER method.

## Conclusions

The connectivity feature map (CFM) of the EEG signal, comprising signals from pairs of channels, is an effective 2D representation of the EEG signal for emotion recognition using DL. Existing connectivity methods construct mostly symmetric CFM having redundant values. As redundant feature values are not effective in improving the DL model’s performance, minimizing redundancy as well as information enhancement in CFM is investigated in this study for improved EEG-based emotion recognition. This study has proposed a fused CFM construction method integrating two different triangular sections from two CFMs produced by two different individual methods. Thus, the fused CFM holds more information than a traditional CFM. Specifically, four widely used connectivity methods (i.e., PCC, PLV, MI, and TE) have been used to produce four base CFMs, and then six fused CFMs (i.e., PCC + PLV, PCC + MI, PCC + TE, PLV + MI, PLV + TE, and MI + TE) have been constructed. Considering information-enhanced fused CFM as the main focus of the study, a generic CNN architecture is used for emotion classification from CFMs for simplicity and brevity.

The proposed method with six different fused CFMs has been evaluated on the DEAP benchmark EEG dataset and rigorously compared with four base CFMs based on emotion classification from CFMs by CNN. Due to the distinct properties of base CFMs, performance is found to be different from the fused CFMs. Among the base CFMs, TE was inferior to others; and the fused CFMs with TE (i.e., PCC + TE, PLV + MI, PLV + TE, and MI + TE) were not found so effective in improving performance. Base CFMs by PLV and MI are found to hold connectivity values more vibrant than others. Therefore, the fused CFM with PLV + MI is found to be the most promising in outperforming other individual or fused CFM methods. The outperformance or at least competitiveness of the proposed method to the state-of-the-art reveals the effectiveness of the information enhancement with CFM fusion. Interestingly, as demonstrated by experimental results, CFM fusion does not incur extra training time for CNN. However, the proposed method is found inferior to a few existing methods, which consider shorter segmentation and hybrid DL methods. On the other hand, the performance of the proposed method for cross-subject cases is worse than subject-dependent cases; therefore, it is also a remaining challenging issue to achieve improved cross-subject classification performance with necessary techniques and modifications employment.

Several potential future research scopes appear from the present study. There are three emotional dimensions: Valence, Arousal, and Dominance. This study evaluates the proposed model only for Valence and Arousal dimensions, which can be extended further by combining the Dominance emotional dimension. A CFM construction combing pairs of connectivity methods is found to be effective in this study over the CFMs with individual methods. A challenging but exciting extension of this study might be the different ways to integrate more features inside a fused CFM. A 3D CFM may be a possible extension combining multiple connectivity feature extraction methods (e.g., PCC + PLV + MI) with some innovative fusion techniques.

The innovative technique proposed in this study is expected to produce better outcomes in other EEG-based investigations. The proposed information-enhanced fused CFM is evaluated on the DEAP dataset for emotion recognition in the present study. Specifically, treating CFM fusion as a general information enhancement approach may also be practical for obtaining better outcomes in seizure detection, autism detection, and other EGG-based machine learning studies.

## Data Availability

The data that support the findings of this study are available from DEAP Dataset (https://www.eecs.qmul.ac.uk/mmv/datasets/deap/) but restrictions apply to the availability of these data, which were used under license for the current study, and so are not publicly available. Data are however available from the corresponding author (M. A. H. Akhand, Email: akhand@cse.kuet.ac.bd) upon reasonable request and with permission of DEAP Dataset authority.

## References

[1] Islam MR (2021). Emotion recognition from EEG signal focusing on deep learning and shallow learning techniques. IEEE Access.

[2] Khattak A, Asghar MZ, Ali M, Batool U (2022). An efficient deep learning technique for facial emotion recognition. Multimed. Tools Appl..

[3] Morais, E., Hoory, R., Zhu, W., Gat, I., Damasceno, M., & Aronowitz, H. Speech emotion recognition using self-supervised features. In *ICASSP 2022–2022 IEEE International Conference on Acoustics, Speech and Signal Processing (ICASSP)* 6922–6926 (2022). 10.1109/ICASSP43922.2022.9747870.

[4] Kessous L, Castellano G, Caridakis G (2009). Multimodal emotion recognition in speech-based interaction using facial expression, body gesture and acoustic analysis. J. Multimodal User Interfaces.

[5] Liu X (2019). Emotion recognition and dynamic functional connectivity analysis based on EEG. IEEE Access.

[6] Chen, M., Han, J., Guo, L., Wang, J., & Patras, I. Identifying valence and arousal levels via connectivity between EEG channels. In *2015 International Conference on Affective Computing and Intelligent Interaction, ACII 2015*, 63–69 (2015). 10.1109/ACII.2015.7344552.

[7] Gao Y, Wang X, Potter T, Zhang J, Zhang Y (2020). Single-trial EEG emotion recognition using Granger causality/transfer entropy analysis. J. Neurosci. Methods.

[8] Alarcão SM, Fonseca MJ (2019). Emotions recognition using EEG signals: A survey. IEEE Trans. Affect. Comput..

[9] Maria MA, Akhand MAH, Hossain ABMA, Kamal MAS, Yamada K (2023). A comparative study on prominent connectivity features for emotion recognition from EEG. IEEE Access.

[10] Peya ZJ, Akhand MAH, Srabonee JF, Siddique N (2022). Autism detection from 2D transformed EEG signal using convolutional neural network. J. Comput. Sci..

[11] Akbari H (2022). Recognizing seizure using Poincaré plot of EEG signals and graphical features in DWT domain. Bratisl. Med. J..

[12] Akbari H, Sadiq MT, Payan M, Esmaili SS, Baghri H, Bagheri H (2021). Depression detection based on geometrical features extracted from SODP shape of EEG signals and binary PSO. Trait. Signal.

[13] Miah ASM, Rahim MA, Shin J (2020). Motor-imagery classification using riemannian geometry with median absolute deviation. Electronics.

[14] Moon S-E, Chen C-J, Hsieh C-J, Wang J-L, Lee J-S (2020). Emotional EEG classification using connectivity features and convolutional neural networks. Neural Netw..

[15] Adeli H, Ghosh-Dastidar S (2010). Wavelet-Chaos methodology for analysis of EEGs and EEG sub-bands. Automated EEG-based diagnosis of neurological disorders.

[16] Islam MR (2021). EEG channel correlation based model for emotion recognition. Comput. Biol. Med..

[17] Liu S (2018). Study on an effective cross-stimulus emotion recognition model using EEGs based on feature selection and support vector machine. Int. J. Mach. Learn. Cybern..

[18] Li J, Zhang Z, He H (2018). Hierarchical convolutional neural networks for EEG-based emotion recognition. Cognit. Comput..

[19] Luo Y (2020). EEG-based emotion classification using deep neural network and sparse autoencoder. Front. Syst. Neurosci..

[20] Chen J, Min C, Wang C, Tang Z, Liu Y, Hu X (2022). Electroencephalograph-based emotion recognition using brain connectivity feature and domain adaptive residual convolution model. Front. Neurosci..

[21] Moon, S.-E., Jang, S., & Lee, J.-S. Convolutional neural network approach for EEG-based emotion recognition using brain connectivity and its spatial information. In *2018 IEEE International Conference on Acoustics, Speech and Signal Processing (ICASSP)*, 2556–2560 (2018). 10.1109/ICASSP.2018.8461315.

[22] Niso G (2013). HERMES: Towards an integrated toolbox to characterize functional and effective brain connectivity. Neuroinformatics.

[23] Farashi S, Khosrowabadi R (2020). EEG based emotion recognition using minimum spanning tree. Phys. Eng. Sci. Med..

[24] Wang Z, Hu S-Y, Song H (2019). Channel selection method for EEG emotion recognition using normalized mutual information. IEEE Access.

[25] Zhang R, Wang Z, Liu Y (2022). The research of EEG feature extraction and classification for subjects with different organizational commitment. MATEC Web Conf..

[26] Arnau-González P, Arevalillo-Herráez M, Ramzan N (2017). Fusing highly dimensional energy and connectivity features to identify affective states from EEG signals. Neurocomputing.

[27] Mert A, Akan A (2018). Emotion recognition based on time–frequency distribution of EEG signals using multivariate synchrosqueezing transform. Digit. Signal Process..

[28] Jagodnik M, Bartal A, Yang H, Huang S, Guo S, Sun G (2022). Multi-classifier fusion based on MI-SFFS for cross-subject emotion recognition. Entropy.

[29] Mehmood RM, Bilal M, Vimal S, Lee S-W (2022). EEG-based affective state recognition from human brain signals by using Hjorth-activity. Measurement.

[30] Pane ES, Wibawa AD, Purnomo MH (2019). Improving the accuracy of EEG emotion recognition by combining valence lateralization and ensemble learning with tuning parameters. Cogn. Process..

[31] Yin Z, Liu L, Chen J, Zhao B, Wang Y (2020). Locally robust EEG feature selection for individual-independent emotion recognition. Expert Syst. Appl..

[32] Apicella A, Arpaia P, Mastrati G, Moccaldi N (2021). EEG-based detection of emotional valence towards a reproducible measurement of emotions. Sci. Rep..

[33] Subasi A, Tuncer T, Dogan S, Tanko D, Sakoglu U (2021). EEG-based emotion recognition using tunable Q wavelet transform and rotation forest ensemble classifier. Biomed. Signal Process. Control.

[34] Goshvarpour A, Goshvarpour A (2022). Innovative Poincare’s plot asymmetry descriptors for EEG emotion recognition. Cogn. Neurodyn..

[35] Goshvarpour A, Goshvarpour A (2023). Lemniscate of Bernoulli’s map quantifiers: Innovative measures for EEG emotion recognition. Cogn. Neurodyn..

[36] Moctezuma LA, Abe T, Molinas M (2022). Two-dimensional CNN-based distinction of human emotions from EEG channels selected by multi-objective evolutionary algorithm. Sci. Rep..

[37] Topic A, Russo M (2021). Emotion recognition based on EEG feature maps through deep learning network. Eng. Sci. Technol. an Int. J..

[38] Li Y, Huang J, Zhou H, Zhong N (2017). Human emotion recognition with electroencephalographic multidimensional features by hybrid deep neural networks. Appl. Sci..

[39] Yuvaraj R, Baranwal A, Prince AA, Murugappan M, Mohammed JS (2023). Emotion recognition from spatio-temporal representation of EEG signals via 3D-CNN with ensemble learning techniques. Brain Sci..

[40] Khan MS, Salsabil N, Alam MGR, Dewan MAA, Uddin MZ (2022). CNN-XGBoost fusion-based affective state recognition using EEG spectrogram image analysis. Sci. Rep..

[41] Wei C, Chen L-l, Song Z-z, Lou X-g, Li D-d (2020). EEG-based emotion recognition using simple recurrent units network and ensemble learning. Biomed. Signal Process. Control.

[42] Liu L, Ji Y, Gao Y, Li T, Xu W (2022). A data-driven adaptive emotion recognition model for college students using an improved multifeature deep neural network technology. Comput. Intell. Neurosci..

[43] Song T, Zheng W, Song P, Cui Z (2018). EEG emotion recognition using dynamical graph convolutional neural networks. IEEE Trans. Affect. Comput..

[44] Asadzadeh S, Rezaii TY, Beheshti S, Meshgini S (2022). Accurate emotion recognition using Bayesian model based EEG sources as dynamic graph convolutional neural network nodes. Sci. Rep..

[45] Khosrowabadi R (2018). Stress and perception of emotional stimuli: Long-term stress rewiring the brain. Basic Clin. Neurosci..

[46] Petrantonakis PC, Hadjileontiadis LJ (2011). A novel emotion elicitation index using frontal brain asymmetry for enhanced EEG-based emotion recognition. IEEE Trans. Inf. Technol. Biomed..

[47] Bagherzadeh S, Maghooli K, Shalbaf A, Maghsoudi A (2022). Recognition of emotional states using frequency effective connectivity maps through transfer learning approach from electroencephalogram signals. Biomed. Signal Process. Control.

[48] Bagherzadeh S, Maghooli K, Shalbaf A, Maghsoudi A (2022). Emotion recognition using effective connectivity and pre-trained convolutional neural networks in EEG signals. Cogn. Neurodyn..

[49] Chao H, Dong L, Liu Y, Lu B (2020). Improved deep feature learning by synchronization measurements for multi-channel EEG emotion recognition. Complexity.

[50] Jin L, Kim EY (2020). Interpretable cross-subject EEG-based emotion recognition using channel-wise features. Sensors.

[51] Koelstra S (2012). DEAP: A database for emotion analysis; using physiological signals. IEEE Trans. Affect. Comput..

[52] Candra, H. *et al.*, Investigation of window size in classification of EEG-emotion signal with wavelet entropy and support vector machine. In *2015 37th Annual International Conference of the IEEE Engineering in Medicine and Biology Society (EMBC)*, 7250–7253 (2015). 10.1109/EMBC.2015.7320065. 10.1109/EMBC.2015.7320065 26737965

[53] Delorme A, Makeig S (2004). EEGLAB: An open source toolbox for analysis of single-trial EEG dynamics including independent component analysis. J. Neurosci. Methods.

[54] Siirtola P, Tamminen S, Chandra G, Ihalapathirana A, Röning J (2023). Predicting emotion with biosignals: A comparison of classification and regression models for estimating valence and arousal level using wearable sensors. Sensors.

[55] Shannon CE (1948). A mathematical theory of communication. Bell Syst. Tech. J..

[56] Akhand MAH, Ahmed M, Rahman MMH, Islam MM (2018). Convolutional neural network training incorporating rotation-based generated patterns and handwritten numeral recognition of major Indian scripts. IETE J. Res..

[57] Akhand MAH (2021). Deep learning fundamentals: A practical approach to understanding deep learning methods.

[58] Akhand MAH, Rahat-Uz-Zaman M, Hye S, Kamal MAS (2023). Handwritten numeral recognition integrating start-end points measure with convolutional neural network. Electronics.

[59] Kingma, D. P. & Ba, J. Adam: A method for stochastic optimization. arXiv:1412.6980. 10.48550/arXiv.1412.6980 (2014).

[60] Li R, Ren C, Zhang X, Hu B (2022). A novel ensemble learning method using multiple objective particle swarm optimization for subject-independent EEG-based emotion recognition. Comput. Biol. Med..

